# Short-term efficacy of music therapy combined with α binaural beat therapy in disorders of consciousness

**DOI:** 10.3389/fpsyg.2022.947861

**Published:** 2022-09-06

**Authors:** Zi-Bo Liu, Yan-Song Liu, Long Zhao, Man-Yu Li, Chun-Hui Liu, Chun-Xia Zhang, Hong-Ling Li

**Affiliations:** ^1^Department of Endocrinology, Second Hospital of Hebei Medical University, Shijiazhuang, China; ^2^The Second Department of Rehabilitation, Second Hospital of Hebei Medical University, Shijiazhuang, China

**Keywords:** disorders of consciousness, music therapy, binaural beat therapy, wakefulness promotion, clinical efficacy

## Abstract

**Objective:**

To investigate the short-term effect of music therapy combined with binaural frequency difference therapy on patients with consciousness disorder.

**Materials and methods:**

Ninety patients with definite diagnosis of disorders of consciousness (DOC) were selected. These patients were randomly divided into control group, experiment 1 group and experiment 2 group, with 30 patients in each group. The control group was treated with routine clinical treatment and rehabilitation. In experiment 1 group, music therapy was added to the control group. In experimental group 2, music therapy combined with binaural α frequency difference therapy was added to the control group. All patients were assessed before and after 30 treatments. The assessment items included Glasgow Coma Scale (GCS), Coma Recovery Scale revised (CRS-R), electroencephalogram (EEG), upper somatosensory evoked potential (USEP), and brainstem auditory evoked potential (BAEP).

**Results:**

Before treatment, there were no significant differences in GCS score, CRS-R score, USEP, BAEP, and EEG scores among the three groups (*P* > 0.05). After 30 times of treatment, GCS score, CRS-R score, USEP, BAEP, and EEG scores in 3 groups were significantly higher than those before treatment (*P* < 0.05), and experimental group 2 >experimental group 1 >control group (*P* < 0.05). And the consciousness rate of experimental group 2 was better than experimental group 1, experimental group 1 was better than the control group and the difference was statistically significant (*P* < 0.05).

**Conclusion:**

Music therapy combined with binaural α frequency difference therapy is more effective in stimulating DOC patients.

## Introduction

### Clinical status of disorders of consciousness

Disorders of Consciousness (DOC) is a state caused by focal or massive brain injury. Common causes of DOC include Traumatic Brain Injury (TBI), stroke, encephalitis, and anoxia following a heart attack. The most common of these causes is TBI. There are at least 1.7 million new cases of TBI in the United States and at least 1.5 million new cases of TBI in Europe each year, and the incidence of TBI is increasing year by year (Kalra et al., 2022). Accurate statistics are not available for this in China, but TBI patients should be a large group in China (Cheng and Hu, 2018).

Some treatments are currently available for DOC patients. There are a number of treatments available for DOC. Medications include amantadine (Giacino et al., 2012) and zolpidem (Whyte et al., 2014). Non-pharmacological treatments include many neuromodulation therapies, such as transcranial direct current stimulation (tDCS), deep brain stimulation (DBS), etc.(Thibaut et al., 2019). Nevertheless, the treatment of DOC remains a major clinical challenge.

Music Therapy (MT) refers to the use of music as a medium to positively influence a patient’s health by listening to, playing, and making music (Wang and Agius, 2018). When music was used for treatment without support from music therapists, this system was called a form of music therapy. Some studies have reported that music can activate brain areas responsible for emotion, cognition, memory and movement (Carrière et al., 2020; Li et al., 2020). In both ears, they may perceive a beat frequency that originates in the superior olivary nucleus, which is the first nucleus in the auditory pathway to receive bilateral input. Some research suggests that exposure to these binaural beats may lead to changes in mood, cognition, and consciousness (Orozco Perez et al., 2020). In recent years, music therapy has begun to be used for patients with DOC (Liu and Li, 2021), and there has been substantial evidence of the clear efficacy of music for patients with DOC (Magee and O’Kelly, 2015). Binaural Beat Therapy (BBT) is a method that involves the input of two different frequencies of audio stimuli to the left and right ears. It can cause a difference in excitatory potentials between the left and right ears and induce a corresponding difference in brain waves to neutralize them. This method was used to induce brain waves through music, cause changes in the subject’s electroencephalogram (EEG), and thus alter the patient’s cognitive, emotional and other conditions (Zhou et al., 2015). Current research on BBT has focused on β beat frequency (14–30 HZ) and θ beat frequency (4–7 HZ), and most of them have been used to improve the cognitive status of the subjects. Fewer studies have been conducted on the application of α beat frequency (8–13 HZ) music, and few studies have used the BBT in the field of DOC. Since α beat is the main rhythm of EEG in normal adult awake and quiet with eyes closed, we speculate that α beat frequency music may have some wake-promoting effect on DOC patients.

The purpose of this study was to combine α beat music with patients’ favorite music in order to investigate the efficacy of different types of music for patients with DOC in a short term.

## Materials and methods

### Clinical information

#### Research subjects

Patients with DOC who visited various departments of our hospital from September 2020 to January 2021, with clear diagnosis and meeting the inclusion criteria, were selected for this study.

### Inclusion and exclusion criteria

#### Inclusion criteria

(1) Patients with GCS scores of 3–8, as well as VS and MCS patients who met the diagnostic criteria. The diagnosis of VS was in accordance with the diagnostic criteria of vegetative state developed by the Emergency Medicine Branch of the Chinese Medical Association in 1996 (Chinese Society of Emergency Medicine Research Group on Disorders of Consciousness, 1996), and the diagnosis of MCS was in accordance with the diagnostic criteria of MCS proposed by Giacino et al. (2002). (2) The age was between 18 and 80 years old. (3) Informed consent of the patient’s family. (4) Duration of disease within 3 months. (5) No sedative drugs were used after the start of the experiment.

#### Exclusion criteria

(1) Those with unstable vital signs during the experiment. (2) Those with severe cardiac arrhythmias or pacemaker implants. (3) Hearing impairment due to ear disease. (4) The patient’s family did not agree. (5) Those who died or were transferred to other hospital during the experiment.

### Main instruments applied

➀ Empower Music Therapy (EMT) headphones

Production company: Hebei Caiwen Technology Co.

➁ Digital EEG

Production Company: Shanghai Nuocheng Electric Co.

➂ VitalStim Plus Electrotherapy System

Model: 5900 model.

Production Company: Beijing Chengkang Jiye Medical Equipment Co.

➃ Electroencephalographic bionic electrical stimulator

Model: NK-IA05.

Production company: Shijiazhuang Du Kang Medical Equipment Co.

➄ Medical three-compartment seven-door air-pressurized hyperbaric oxygen chamber

Model: YC3200/0.3-22V II.

Production Company: Yantai Binglun Hyperbaric Chamber Co.

(6) Intelligent active and passive training system for lower limbs

Production Company: Beijing Baodahua Rehabilitation Equipment Technology Co.

## Research methods

### Study grouping

A total of 90 patients with DOC who visited the departments of the Second Hospital of Hebei Medical University from December 2020 to January 2022 and met the inclusion criteria were divided into control, experimental 1 and experimental 2 groups using the random number table method, with 30 patients in each group. Statistical analysis of the basic information of the patients in the three groups showed no statistical difference between the three groups (*P* > 0.05). See Table 1.

**TABLE 1:** Comparison of basic conditions of the three groups before treatment.

Group	number	Age (years)	Gender		Course of disease	Type of disease	
			M	F		Tra	Non-tra
CG	30	58.03 ± 7.23	16	14	34.2 ± 2.96	8	22
EG1	30	60.61 ± 11.0	18	12	35.2 ± 3.84	10	20
EG2	30	59.93 ± 10.05	17	13	36.1 ± 4.63	10	20

Control group *n* = 30, experimental group 1 *n* = 30, experimental group 2 *n* = 30.

CG, control group; EG1, experimental group 1; EG2, experimental group 2; M, male; F, female; Tra, trauma; Non-tra, non-trauma.

### Treatment methods

**FIGURE 1:**
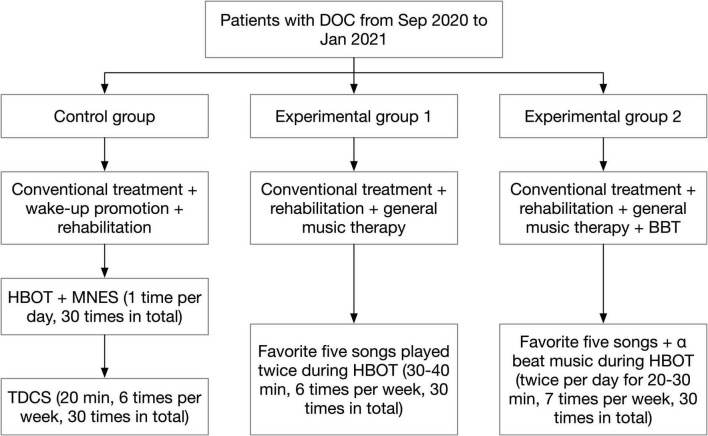
Flow diagram of group assignment. BBT, binaural beat therapy; DOC, disturbance of consciousness; HBOT, hyperbaric oxygen therapy; MNES, median nerve stimulation; TDCS, transcranial direct current stimulation.

After admission, patients were routinely treated and cared for by the first-visit unit according to their condition. This included treatment with medication or surgery, anti-infection therapy, correction of electrolyte balance disorders and dehydration to lower cranial pressure, continuous monitoring of blood pressure, ECG and oxygen saturation, and nursing measures such as body turning and sputum evacuation. After the patient’s condition was stabilized, a rehabilitation physician would consult with the DOC patient and developed a rehabilitation treatment plan according to the patient’s condition. The rehabilitation therapist and rehabilitation physician would also closely observe the patient’s condition during the post-treatment process and adjust the rehabilitation treatment plan in a timely manner when there were changes in the condition. The group process was shown in Figure 1.

#### Control group

On the basis of conventional treatment, patients were provided with wake-up promotion and rehabilitation treatment according to their conditions and functional disorders. The wake-up promotion treatment included: ➀ Hyperbaric Oxygen Therapy (HBOT): the treatment was performed with 2.0 ATA pressure, pressurization for 25 min, stabilization for 70 min. During the stabilization process, pure oxygen was administered with the mask on for 30 min, then the mask was removed to rest for 10 min, and finally the oxygen was administered with the mask on for 30 min. The pressure was decompressed to normal pressure for 25 min after stabilization, and the patient was discharged from the chamber. The treatment was performed 1 time per day, 7 times per week, and 30 times in total. ➁ Median Nerve Stimulation (MNES): MNES was performed while the patient was undergoing HBOT. Before pressurizing the oxygen chamber, two electrode sheets were placed side by side and attached to the patient’s right side 2 cm above the transverse wrist line, and two electrode wires of the median nerve stimulator were connected to the electrode sheets. The stimulation intensity was adjusted to 15 mA to cause slight flexion of the patient’s right middle finger. The stimulation time was 2 h. The treatment was performed 1 time per day, 7 times per week, and 30 times in total. ➂ TDCS: The treatment site was the dorsolateral aspect of the left prefrontal lobe, the anode was placed on the dorsolateral aspect of the left prefrontal lobe, and the cathode was placed on the right shoulder; the current intensity was set to 2.0 mA, each treatment was 20 min, 6 times a week, for a total of 30 treatments. Rehabilitation treatment included: ➀ Good limb position placement. ➁ Passive activity. ➂ Neuromuscular Electrical Stimulating (NMES). ➃ Electrical stimulation of swallowing muscle groups.

#### Experiment group 1

General music therapy was added for patients on the basis of conventional treatment and the wake-up promotion and rehabilitation therapy received by the control group. In fact, we used a system that delivered a form of music therapy without the support of a music therapist. After the patients were enrolled in the group, the rehabilitation physician asked the patients’ family members about patients’ favorite music and singers before the onset of illness (if the patients did not have the habit of listening to music, popular songs that fit their age group were selected), and a total of 5 songs that the patients liked were collected. Subsequently, the patient’s favorite songs were downloaded and transferred to the empowering music headphones produced by Hebei Caiwen Technology Co. The headphones were worn for the patients while they were undergoing HBOT, and the 5 music songs were played twice in a cycle of 30–40 min each time, 6 times/week, for a total of 30 times treatment.

#### Experiment group 2

The patients received music therapy combined with BBT in addition to the conventional treatment and the wake-up promotion and rehabilitation treatment received by the control group. The rehabilitation physician asked the patients’ family members about patients’ favorite music and singers before the onset of illness, and found out 5 pieces of music that the patient loved the most. The music was downloaded through the Internet. Music and BBT were merged during a same single session and played simultaneously. The five pieces of music were then edited together using the audio editing software Dominic Mazzoni Audacity 2.33 (United States MA 02110-1301) provided by Hebei Caiwen Technology Co, and the α beat music provided by Hebei Caiwen Technology Co., Ltd. was used as background music and merged with the patient’s favorite 5 music tracks to create individualized wake-up-promoting music. EMT headphones were used to play the music twice a day for 20–30 min while the patient was undergoing HBOT, 7 times/week, for a total of 30 times treatment.

#### Notes

During the rehabilitation treatment, if the patient developed uncomfortable symptoms such as heavy sweating, rapid heart rate and pale face, the treatment should be stopped immediately and the cause should be identified together with the doctor in charge to ensure that the patient’s condition would not deteriorate as a result of the rehabilitation treatment. If the patient’s symptoms disappeared after a few minutes of rest, the rehabilitation treatment could be attempted again; if the above-mentioned uncomfortable symptoms still appeared after the rehabilitation treatment was reintroduced, the rehabilitation treatment was stopped on the same day and a new rehabilitation treatment plan was discussed and formulated by the rehabilitation physician, rehabilitation therapist and the patient’s supervising doctor, and the rehabilitation treatment was restarted on the next day.

Since music therapy and median nerve electrical stimulation were performed simultaneously with HBOT, the only people accompanying the patient in the cabin were the patient’s family members, so the patient’s family members should be educated before entering the cabin and report the patient’s discomfort to the medical staff outside the cabin as soon as it was detected. The volume level of music should be noted during the music therapy treatment. The volume of the empowering music headphones was divided into 10 levels, with level 4 being the tolerable and more appropriate volume for normal adults, and a higher volume may damage the patient’s hearing.

### Observed indicators

As soon as the rehabilitation physician determined that the patient was ready to be enrolled, the patient was assessed immediately with the Glasgow Coma Scale (GCS) (Bodien et al., 2021), Coma Recovery Scale-Revised (CRS-R) (Ferraro et al., 2020), EEG (Scarpino et al., 2020), upper somatosensory evoked potential (USEP) (Moshayedi et al., 2019), and Brainstem Auditory Evoked Potential (BAEP) (Zhang et al., 2015). Classification was carried out according to GCS score (Bodien et al., 2021): (1) Basically cured: the patient had clear consciousness with a GCS score of 15 points. (2) Significant effectiveness: the patient’s consciousness state improved significantly with a GCS score of ≥12. (3) Effectiveness: the patient’s consciousness was improved with a GCS score of ≥9. (4) Invalid: there was no improvement in patients’ consciousness with no improvement or even decrease in GCS score. Only a GCS score of ≥9 after treatment can identify the improvement of consciousness, and the improvement rate of consciousness is the ratio of the number of (effective + significantly effective + basically cured) people and the total number of people. EEG classification was based on the method by Hockaday et al. (1965): Grade I was EEG with α rhythm as the basic rhythm, which was close to normal adult EEG and rated as 3. For grade II, θ wave is dominant, with less δ wave, which is rated as 2. Grade III showed that the EEG was dominated by δ wave and no other wave appeared, which was rated as 1. Grade IV showed that the EEG rhythm almost disappeared and the waveform was flat, which was rated as 0. These assessments were repeated on the day after the last treatment of 30 sessions. The pre-treatment and post-treatment assessments were performed by the same person.

### Statistical analysis methods

SPSS 21.0 software was used for statistical analysis of the experimental results. The measurement data were expressed as mean ± standard deviation (x ± s). If the data were normally distributed and had homogeneity of variance, ANOVA was used to compare the three groups of data, and if the data were not normally distributed and did not have homogeneity of variance, non-parametric tests were used. The test level was α = 0.05, and the difference was statistically significant if P was less than 0.05.

## Results

### Glasgow coma scale score

Before treatment, there was no statistical difference in GCS scores among patients in the control group, experimental group 1 and experimental group 2 (*P* > 0.05). After 30 treatments, the GCS score was (8.30 ± 3.32) in the control group, (10.70 ± 3.13) in the experimental group 1, and (12.50 ± 2.49) in the experimental group 2. All three groups of patients improved their GCS scores after treatment compared with the scores before treatment (*P* < 0.05), and the GCS scores in the experimental group 2 were greater than those in the experimental group 1, and the GCS scores in the experimental group 1 were greater than those in the control group, with statistically significant differences (*P* < 0.05). See Table 2.

**TABLE 2:** Glasgow Coma Scale (GCS) scale score of patients in the three groups ($\bar{\text{x}}$ ± s, control group *n* = 30, experimental group 1 *n* = 30, experimental group 2 *n* = 30).

Group	Before treatment	After treatment
CG	5.43 ± 1.76^➀^	8.30 ± 3.32^➁^
EG1	5.50 ± 1.85^➀^	10.70 ± 3.13^➁ ➂^
EG2	5.53 ± 1.66^➀^	12.50 ± 2.49^➁ ➃^

^➀^*P* > 0.05 control group, Experimental group 1 and experimental group 2 control group before treatment; ^➁^*P* < 0.05 Experimental group 1 and experimental group 2 and control group after treatment; ^➂^*P* < 0.01 Experimental group 1 compared with control group after treatment; ^➃^*P* < 0.01 Experimental group 2 compared with experimental group 1 and control group after treatment.

### Coma recovery scale revised score

Before treatment, there was no statistical difference in the CRS-R scores of patients in the control group, experimental group 1 and experimental group 2 (*P* > 0.05). After 30 treatments, the CRS-R score was (11.20 ± 2.28) in the control group, (12.60 ± 2.08) in the experimental group 1, and (14.57 ± 1.92) in the experimental group 2. All three groups of patients improved their CRS-R scores after treatment than those before (*P* < 0.05), and the CRS-R scores in the experimental group 2 were greater than those in the experimental group 1, and the CRS-R scores in the experimental group 1 were greater than those in the control group, and the differences were statistically significant (*P* < 0.05). See Table 3.

**TABLE 3:** Coma Recovery Scale revised (CRS-R) scale score of patients in the three groups ($\bar{\text{x}}$ ± s, control group *n* = 30, experimental group 1 *n* = 30, experimental group 2 *n* = 30).

Group	Before treatment	After treatment
CG	9.17 ± 2.21^➀^	11.20 ± 2.28^➁^
EG1	9.67 ± 1.95^➀^	12.60 ± 2.08 ^➁ ➂^
EG2	9.90 ± 2.25^➀^	14.57 ± 1.92^➁ ➃^

^➀^*P* > 0.05 control group, Experimental group 1 and experimental group 2 control group before treatment; ^➁^*P* < 0.05 Experimental group 1 and experimental group 2 and control group after treatment; ^➂^*P* < 0.01 Experimental group 1 compared with control group after treatment; ^➃^*P* < 0.01 Experimental group 2 compared with experimental group 1 and control group after treatment.

### USEP

Before treatment, most patients were rated as grade 2 and 3 of USEP score and there was no statistical difference in USEP scores among the control group, experimental group 1, and experimental group 2 (*P* > 0.05). The USEP scores of all three groups improved after treatment compared with those before (*P* < 0.05), and the experimental group 2 had less proportion of USEP grade 2 than those of the experimental group 1, and the experimental group 1 had less proportion of USEP grade 3 than those of the control group, and the difference was statistically significant (*P* < 0.05). See Table 4.

**TABLE 4:** Grade of sensory evoked potential of the upper limbs of patients in three groups ($\bar{\text{x}}$ ± s, control group *n* = 30, experimental group 1 *n* = 30, experimental group 2 *n* = 30).

Group	*n*	Time	I	II	III	IV
CG	30	BT^➀^	3	11	15	1
		AT^➁^	5	17	16	2
EG1	30	BT^➀^	2	12	14	2
		AT^➁ ➂^	9	19	1	1
EG2	30	BT^➀^	3	11	14	2
		AT^➁ ➃^	17	13	0	0

^➀^*P* > 0.05 control group, Experimental group 1 and experimental group 2 control group before treatment; ^➁^*P* < 0.05 Experimental group 1 and experimental group 2 and control group after treatment; ^➂^*P* < 0.01 Experimental group 1 compared with control group after treatment; ^➃^*P* < 0.01 Experimental group 2 compared with experimental group 1 and control group after treatment. BT, before treatment; AT, after treatment.

### Brainstem auditory evoked potential

Before treatment, most patients were rated as grade 2 and 3 of BAEP score and there was no statistical difference in BAEP scores among patients in the control group, experimental group 1 and experimental group 2 (*P* < 0.05). The BAEP scores of all three groups improved after treatment compared with those before (*P* < 0.05), and the experimental group 2 had less proportion of BAEP grade 2 than those of the experimental group 1, and the experimental group 1 had less proportion of BAEP grade 3 than those of the control group, and the difference was statistically significant (*P* < 0.05). See Table 5.

**TABLE 5:** Three groups of patients brain stem evoked potential grading score ($\bar{\text{x}}$ ± s, control group *n* = 30, experimental group 1 *n* = 30, experimental group 2 *n* = 30).

Group	*n*	Time	I	II	III	IV
CG	30	BT^➀^	1	20	8	1
		AT^➁^	5	16	9	0
EG1	30	BT^➀^	0	15	14	0
		AT^➁ ➂^	8	20	2	0
EG2	30	BT^➀^	2	16	10	0
		AT^➁ ➃^	17	12	1	0

^➀^*P* > 0.05 control group, Experimental group 1 and experimental group 2 control group before treatment; ^➁^*P* < 0.05 Experimental group 1 and experimental group 2 and control group after treatment; ^➂^*P* < 0.01 Experimental group 1 compared with control group after treatment; ^➃^*P* < 0.01 Experimental group 2 compared with experimental group 1 and control group after treatment. BT, before treatment; AT, after treatment.

### Electroencephalogram

Before treatment, most patients were rated as grade 2 and 3 of EEG score and there was no statistical difference in EEG scores among patients in the control group, experimental group 1 and experimental group 2 (*P* > 0.05). The EEG scores of patients in all three groups improved after treatment compared with those before (*P* < 0.05), and the experimental 2 group had less proportion of EEG grade 2 than those in the experimental group 1, and the experimental group 1 had less proportion of EEG grade 3 than those in the control group, with statistically significant differences (*P* < 0.05). See Table 6.

**TABLE 6:** Electroencephalogram (EEG) grading scores of the patients in the three groups ($\bar{\text{x}}$ ± s, control group *n* = 30, experimental group 1 *n* = 30, experimental group 2 *n* = 30).

Group	*n*	Time	I	II	III	IV
CG	30	BT^➀^	2	13	13	2
		AT^➁^	2	19	9	0
EG1	30	BT^➀^	2	11	15	2
		AT^➁ ➂^	7	19	4	0
EG2	30	BT^➀^	1	14	13	2
		AT^➁ ➃^	15	13	2	0

^➀^*P* > 0.05 control group, Experimental group 1 and experimental group 2 control group before treatment; ^➁^*P* < 0.05 Experimental group 1 and experimental group 2 and control group after treatment; ^➂^*P* < 0.01 Experimental group 1 compared with control group after treatment; ^➃^*P* < 0.01 Experimental group 2 compared with experimental group 1 and control group after treatment. BT, before treatment; AT, after treatment.

### Clinical efficacy

In the control group, 2 patients were basically cured, 6 patients were treated significantly effectively, 2 patients were treated effectively, and the rate of improvement of consciousness was 33.33%. In the experimental group 1, 7 patients were basically cured, 7 patients were treated significantly effectively, 5 patients were treated effectively, and the rate of improvement of consciousness was 63.33%. In the experiment group 2, 6 patients were basically cured, 16 patients were treated significantly effectively, 4 patients were treated effectively, and the rate of improvement of consciousness was 86.7%. Comparing the three groups, the improvement rate of consciousness in the experimental group 2 was higher than that in the experimental group 1, and the improvement rate of consciousness in the experimental 1 group was higher than that in the control group, and the difference was statistically significant (*P* < 0.05). See Table 7.

**TABLE 7:** Clinical efficacy of control group and experimental group.

Group	Basically cured	Significantly effectively	Significantly effectively	Ineffective
Control group	2	6	2	20
Experimental group 1	7	7	5	11
Experimental group 2	6	16	4	4

*P* < 0.05 Control group, experimental group 1 and experimental group 2 after treatment.

## Discussion

Research on DOC has progressed rapidly in recent decades, and the diagnosis and classification of DOC have been gradually refined, and a variety of treatments are available, but there is still a lack of treatments with outstanding effects. The number of patients with DOC is increasing year by year, and any emerging treatment would be of great help to patients with DOC and their families.

### Efficacy of conventional treatment for patients with DOC

All 3 groups of patients were treated with conventional treatments, including tDCS, MNES, and HBOT. The results of this experiment showed that the electrophysiological and behavioral indices of the control group after 30 sessions were better than those before treatment, indicating that conventional treatment also had some wake-promoting effect on DOC patients. It can also result from the spontaneous recovery of neurons because our included population had a duration of disease within 3 months. At an early stage, the damage to the nervous system is mild and reversible. Although there is no evidence that nerves can regenerate, the dysfunction can be compensated for by the remaining intact areas.

Hyperbaric Oxygen Therapy has long been shown by many scholars to have wake-promoting efficacy. Affected brain tissue in DOC patients is in a hypoxic state and neurons begin to metabolize anaerobically. Under continuous hypoxia, neurons are no longer able to maintain metabolic homeostasis and oxygen free radicals accumulate and degrade cell membranes, which will eventually lead to cell death (Ikeda and Long, 1990). HBOT has been shown to reduce the adverse effects of hypoxia on neural tissue (Robertson et al., 1989). It can increase the oxygen supply to brain tissue and reduce brain edema, and reduce oxidative stress and improves mitochondrial function in damaged brain tissue (Hills, 1999; Wang et al., 2016), thus improving the state of consciousness of DOC patients.

Transcranial direct current stimulation has also been used for the treatment of DOC in recent years. The tDCS emits a weak direct current, which passes through the liner to the skull. Neurons in the corresponding brain regions are stimulated by the direct current and undergo changes in excitability and produce certain electrical activity. tDCS treatment of DOC is based on the mechanism that the anode can increase the excitability of the subject’s cortex and induce increased connectivity between different brain regions, affecting the interaction between different brain regions. For the treatment of DOC, the anode of tDCS is placed in the left dorsolateral prefrontal lobe, which is the center in charge of higher cognitive functions such as attention and memory, when it can have maximum wake-promoting effects on DOC patients (Aloi et al., 2022).

The median nerve is thought to be the gateway to the CNS, and stimulation of the median nerve transmits nerve impulses to the ascending arousal system of the brainstem to increase the excitability of this brain region (Cooper et al., 2005). In addition, it has been found that stimulation of the median nerve increases the expression of hypothalamic orexin-A and its receptors, which is an excitatory neurotransmitter that increases CNS excitability and promotes arousal (Zhong et al., 2015).

### The efficacy of music therapy on DOC patients

Music therapy is a relatively new treatment compared to drugs and other neuromodulation therapies, which was first applied to five TBI patients in 1990 by Aldridge et al. (1990) and found that music improved the consciousness of DOC patients to some extent. A possible mechanism for music therapy to improve consciousness is the promotion of CNS remodeling (Vik et al., 2018). It has been confirmed that music stimulation can promote processes such as dendritic reoccurrence and myelin remodeling in DOC patients (Schlaug et al., 1995). (Vik et al., 2019) also found that music therapy improved behavioral and cognitive function in DOC patients, which may be due to the activation of the medial orbitofrontal cortex in DOC patients under music stimulation. The medial orbitofrontal cortex is affiliated with the prefrontal cortex and is involved in the regulation of cognitive function (Rolls, 2014). In addition to inducing CNS remodeling, the mechanism of music therapy for DOC also includes alleviating the patient’s primary lesion. Regardless of the etiology, patients experience inflammation and oxidative stress in the local brain tissue of the primary lesion (Defalque et al., 1999; Hermann et al., 2019). Musical stimulation has been shown to reduce the inflammatory response in CNS lesions and inhibit responses such as oxidative stress (Yamasaki et al., 2012).

Patients in the experimental group 1 showed improvement in behavioral and electrophysiological indices compared to the control group. There was no statistical difference between the baseline data of the two groups, and the improvement may be related to music therapy. The efficacy of music therapy for DOC patients has been confirmed by many scholars. O’kelly et al. (2013) performed music for DOC patients in the field using musical instruments. All of the above studies have shown clear efficacy. We selected five complete pieces of music that patients love, so that they can listen to their favorite music in its entirety. Studies have shown that people are most sensitive to their own names, which are the most frequent sound stimuli in their daily lives. The patient’s favorite music is also most likely to be the most frequently listened to music, so we selected the patient’s favorite music as the medium of music therapy.

A larger number of patients were selected for our study and a novel way of editing music was chosen. The findings of this study are consistent with previous studies: patients’ favorite music can have a wake-promoting effect on them.

### The efficacy of music therapy combined with binaural beat therapy on DOC patients

The BBT method is mostly applied to regulate human cognition. The innovation of this experiment lies in the use of α beat frequency music for DOC patients. Some studies have reported that binaural beat frequency stimulation in the low frequency band (including δ and θ frequency bands) significantly suppresses anxiety and promotes rapid sleep (Le Scouarnec et al., 2001), and binaural beat frequency stimulation in the high frequency band (β frequency band) significantly enhances memory, attention, alertness, etc. in subjects (Lane et al., 1998). Fewer studies have been conducted on α beat frequency, and this experiment innovatively used α beat frequency music for DOC patients and observed good efficacy.

The behavioral and electrophysiological indexes were improved in experiment group 2 compared with experiment group 1 and control group. It revealed that the combination of α beat frequency music with patients’ favorite music had stronger therapeutic effect on DOC patients. α rhythm is a significant rhythm that can be detected in the EEG of normal adults during awake relaxation with eyes closed, we speculated that if the CNS perceives this frequency of beat frequency may have a certain effect on promoting wakefulness, so the beat frequency music used in this experiment was α beat frequency music. Numerous studies have confirmed that C patients. α rhythm is a significant rhythm that can be detected iory (Basar, 2012). Some scholars have used PET and EEG to conclude that the occipital α rhythm is the result of the interaction of cortical and thalamic neurons, which originate from a subtype of thalamic pacemaker cells; such cells discharge at α beat frequency and are coordinated by electrically active hyperpolarization-activated cyclic nucleotide-gated channels and acetylcholine-activated calcium channels (Sharma and Nadkarni, 2020). When the CNS senses the stimulation of α beat frequency music, it may cause the excitatory discharge of the above-mentioned cells, thus causing changes in the EEG in DOC patients. We played the α beat music as background music together with 5 favorite music clips to the patients in the experimental group 2. The α beat music combined with the favorite music of the patients synergized the wake-promoting effect on the patients. As a result, the behavioral and electrophysiological indices of patients in experiment group 2 were improved compared with the other two groups.

### Occurrence of adverse events

Many clinical trials using music therapy have found no adverse events associated with music therapy, and music therapy is generally a safe treatment. No adverse events such as increased blood pressure, increased heart rate, or seizures occurred in this trial.

### Shortcomings of this experiment and future research directions

Music therapy has been gradually and widely used in the treatment of DOC, but the binaural beat frequency method has not yet begun to be applied to the treatment of DOC on a large scale in clinical practice. In this experiment, we initially explored the efficacy, feasibility, and safety of music therapy combined with BBT. However, our study has many shortcomings: ➀ the number of patients included in our study was relatively small, and future multicenter, large-scale clinical trials could be conducted to make the experimental data more credible. ➁ Due to the time limitation of the study, we were not able to follow up the patients, and the patients could be followed up after a certain period of time to assess the differences in patient outcomes between groups after discharge. In addition, we did not conduct a dynamic monitoring of EEG when patients listened to music and examine the signs of consciousness due to study design and financial issue (Wannez et al., 2017). Therefore, further in-depth studies with comprehensive assessment are warranted to elucidate the mechanism underlying this improved efficacy. ➂ This experiment initially found that α beat frequency music may have some wake-up-promoting effect on DOC patients, but the specific mechanism of it could not be elucidated, and it could be elucidated through basic experiments in the future. ➃ In this experiment, we did not group DOC patients with different etiologies, and in the future, we can group DOC patients with different etiologies to observe the therapeutic effect of music therapy combined with α beat frequency therapy for different etiologies and further elucidate the mechanism.

## Conclusion

In this experiment, 90 patients who met the inclusion and exclusion criteria were randomly divided into control group, experimental group 1 and experimental group 2. The control group was treated with conventional clinical therapy and care; the experimental group 1 was treated with general music therapy on the basis of the control group; the experimental group 2 was treated with α beat frequency therapy combined with music therapy on the basis of the control group. The behavioral and electrophysiological assessments of the patients before and after treatment showed that the combination of α beat frequency therapy and music therapy could maximize the wakefulness-promoting effect on DOC patients. This experiment enabled us to explore a new method for treating DOC, but the mechanism and course of treatment need to be further studied for future large-scale application to DOC patients.

## Data availability statement

The original contributions presented in this study are included in the article/supplementary material, further inquiries can be directed to the corresponding author.

## Ethics statement

The studies involving human participants were reviewed and approved by the Second Hospital of Hebei Medical University (Ethics Approval NO: 2020-R332). The patients/participants provided their written informed consent to participate in this study.

## Author contributions

Z-BL: conception and design. Y-SL and LZ: administrative support. M-YL and C-HL: provision of study materials or patients. C-XZ and H-LL: collection and assembly of data. Z-BL, LZ, and H-LL: data analysis and interpretation. All authors writing the manuscript approved the final of the manuscript.
